# Are residents of high-walkable areas satisfied with their neighbourhood?

**DOI:** 10.1007/s10389-016-0744-5

**Published:** 2016-08-06

**Authors:** Gerlinde Grasser, Sylvia Titze, Willibald J. Stronegger

**Affiliations:** 1 Institute of Social Medicine and Epidemiology, Medical University of Graz, Universitätsplatz 6/I, 8010 Graz, Austria; 2 Health Management in Tourism, FH JOANNEUM, University of Applied Sciences, Bad Gleichenberg, Austria; 3 Institute of Sport Science, University of Graz, Graz, Austria

**Keywords:** Walkability, Geographic information system, Neighbourhood satisfaction, Adults, Residential neighbourhood

## Abstract

**Aim:**

While the association between walkability and walking for transport has been well established, less is known about the association between walkability and neighbourhood satisfaction. This study aims to examine the direction and strength of the association between objective measures of residential walkability and neighbourhood satisfaction, as well as the differences by sex.

**Subjects and methods:**

Using a cross-sectional study design, outcome data were derived from the representative cross-sectional survey (n = 843) ‘Bicycle-friendly City’ of adults in the city of Graz (Austria). Walkability was measured as gross population density, household unit density, entropy index, proportion of mixed land use, three-way intersection density, four-way intersection density and walkability indices. The outcomes were measured as general neighbourhood satisfaction and neighbourhood satisfaction with the general socio-environmental quality, social cohesion and local infrastructure. Logistic regression analyses were conducted, including age, socio-economic status and place of residence.

**Results:**

Walkability was negatively associated with general neighbourhood satisfaction, neighbourhood satisfaction with general socio-environmental quality and social cohesion. It was positively associated with neighbourhood satisfaction with local infrastructure. Connectivity and the entropy index showed the weakest or no association with the outcomes. The strongest association was between walkability and neighbourhood satisfaction with socio-environmental quality. There were no differences by sex.

**Conclusion:**

These results contribute to the current limited understanding of the association between walkability and neighbourhood satisfaction, especially in a European context. More comparable, longitudinal research would be helpful to determine what impact walkability has on neighbourhood satisfaction and to identify the important mediating factors.

## Introduction

Associations between the walkability of residential neighbourhood and health-related outcomes, especially walking for transport, have been shown in a wide range of studies (Saelens et al. 2003). Objectively measured walkability was defined as “the extent to which characteristics of the built environment and land use may or may not be conducive to residents in the area to walk for either leisure, exercise or recreation, to access services, or to travel to work” (Leslie et al. 2007). Walkability has two fundamental aspects: proximity to destinations and connectivity (Frank et al. 2003; Frumkin et al. 2004). Proximity is determined by density and land use mix (Frumkin et al. 2004; Leslie et al. 2007). Density measures the “quantity of people, households or jobs distributed over a unit of area” (Frumkin et al. 2004). Land use mix can be seen as a complement to density and is “a measure of how many types—offices, housing, retail, entertainment, services, and so on—are located in a given area” (Frumkin et al. 2004). Connectivity, which describes the street linkage among destinations, is based on the design of the street network (Leslie et al. 2007).

One aspect related to walkability and health is neighbourhood satisfaction. Neighbourhood satisfaction seems to be associated with health (Stronegger et al. 2010) and has a strong impact on the decision about where to live (Van Dyck et al. 2011). Residents must be satisfied with walkable neighbourhoods, or people would not want to move there. In other words, the fact that people who live in walkable areas are dissatisfied with their neighbourhoods may indicate that walkability is a potential source of distress or dissatisfaction, which makes this a public health issue that requires further investigation (Frumkin 2003).

Studies on this issue are rare and the available evidence has been mixed. One study in Australia found a positive association between perceived walkability and different aspects of neighbourhood satisfaction (Leslie and Cerin 2008). Studies from the US have shown positive associations between walkability and neighbourhood satisfaction, but only in higher-income areas (Sallis et al. 2009) and only for some aspects of neighbourhood satisfaction (especially with attractiveness and safety), while perceived walkability was negatively associated with socio-environmental quality (Lovejoy et al. 2010). A European study, in contrast, found a negative association between density and neighbourhood satisfaction and no association among land use mix, connectivity and the walkability index and neighbourhood satisfaction (Van Dyck et al. 2011). These differences may be explained by the fact that these studies used different measures of the built environment and of neighbourhood satisfaction or perhaps by differences in the context investigated. Further research on the association between walkability and neighbourhood satisfaction would be desirable, especially for the European context.

Additionally, there is no research done on differences by sex regarding the association between walkability and neighbourhood satisfaction. A review that examined the association between the built environment and walking found associations for men more often than for women (Wendel-Vos et al. 2007). Even though women usually have closer community ties because of their multiple roles, the built environment seems to be less important for their walking behaviour than for the walking behaviour of men. More research is needed on the differences by sex regarding the association between walkability and health-related outcomes and neighbourhood satisfaction (Grasser 2014).

Graz, the second largest city in Austria, can be considered a typical European city and is therefore a suitable context to investigate the possible connection between walkability and neighbourhood satisfaction in Europe. Graz has about 250,000 inhabitants and the median population density is about 2000 inhabitants per square kilometre (Graz 2008, 2016). It has an old town in the centre, which is characterised by small streets, medieval houses and pedestrian areas. As the inner districts have limited space for parking, walking, using public transit and cycling are encouraged. The outer districts, in turn, are characterised by residential areas, including areas with single-family houses, but also semi-detached houses and apartment houses. The Mur river splits Graz into the western and eastern halves. The west side of the river has traditionally been the place of residence for blue collar workers, while the east side of the river, where the old university and most of the cultural heritage sites are located, is considered to be the home of the intellectuals (Grasser 2014). Furthermore, the socio-economic composition of the population differs between West and East. The socio-economic status of the population residing in the western half of the city is lower than that of the population residing in the eastern half (Grasser 2014).

The aim of this study was to contribute to the evidence on the association between objectively measured walkability and subjectively measured neighbourhood satisfaction. It examined the direction and strength of association between measures of residential walkability and neighbourhood satisfaction in Graz, as well as differences by sex.

## Methods

The study is based on a representative cross-sectional telephone survey of the population of Graz aged 15–60 years, which was conducted in autumn 2005 within the research project ‘Bicycle-friendly City’. The protocol was approved by the ethics committee of the local medical university (no. 17-083ex05/06). A random sample was taken from the telephone directory based on the last digit of the telephone numbers (Titze et al. 2008). Details on the survey development and procedures are described elsewhere (Stronegger et al. 2010; Titze et al. 2007, 2008; Grasser 2014). The questionnaire showed acceptable test-retest reliability (Titze et al. 2007). The response rate was 69 % (Titze et al. 2008). Of the 997 participants providing data on walking and cycling for transport, 843 provided a valid residential address and were included in the present analysis.

The questionnaire included ten items concerning the individual satisfaction with the neighbourhood. These ten items were assessed on a five-point rating scale ranging from one (very satisfied) to five (not satisfied at all). A general neighbourhood satisfaction score and three factors were created and dichotomised around the median forming the categories ‘high’ and ‘low’ (Stronegger et al. 2010): general socio-environmental quality (including reputation/appearance, location of the neighbourhood, safety, recreational walking opportunities, environmental quality), social cohesion (including social cohesion, relationship with neighbours) and local infrastructure (including public transport, shops and medical services, recreational and leisure time infrastructure).

The size of the neighbourhood was not further defined in the questionnaire. A study of the perceived size of the neighbourhood has shown that about 95 % of the destinations people walk to are within the 1000 m network buffer area (Smith et al. 2010). Therefore, it is assumed that the perceived walking distance in the questionaire corresponds to the 1000 m network buffer used for the GIS analyses in this study. Additionally, the 1000 m street network buffer was chosen because this scale is the most prevalent one in the literature and the exploration of other scales yielded basically identical results (Grasser 2014).

The study measured walkability using geographic information system (GIS). The measures used were gross population density, household unit density, entropy index, proportion of mixed land use, three-way intersection density, four-way intersection density, IPEN walkability index and the Graz walkability index. The GIS protocol of Forsyth and colleagues (Forsyth et al. 2006) served as the basis for the procedures used. The entropy index ranges from 0 to 1, with 0 representing perfect homogeneity and 1 perfectly heterogeneous land use. The present study used the walkability index based on the recommendations of IPEN but omitted the ‘retail floor area ratio’ because of lack of relevance for a European setting (Van Dyck et al. 2010) such as Graz and because no geodata were available. Additionally, the Graz walkability index was used, which is based on the following formula: (z-score four-way intersections) + (z-score proportion of mixed land use) + (z-score household unit density). Further description of these variables can be found elsewhere (Grasser et al. submitted). Statistics Austria delivered the census data, while the city of Graz and the county of Styria provided the GIS data. The walkability measures were calculated using ESRI® ArcMapTM 10.0 and ESRI® ArcCatalogTM 10.0.

Statistical analyses were performed using the SPSS Statistics 17.0 software for Windows. Logistic regressions were conducted to examine the dependence of the odds of being very satisfied with the neighbourhood with each increase in the walkability indicators. The z-scores of the walkability indicators were calculated and used as continuous variables. The walkability measures were used as the independent variables in the regression analyses, while the dichotomised neighbourhood satisfaction variables were used as the dependent variables. The analyses included age (in nine categories), socio-economic status (a cumulative score based on education, occupation and income and used as a continuous variable) and place of residence (west or east of the river) as potential confounders. The analysis was stratified by sex and the OR and their confidence intervals were compared to identify differences by sex. The results section provides the odds ratios, 95 % confidence intervals, regression coefficient and p-value.

## Results

Table 1 shows that female respondents, respondents with a high socio-economic status and respondents residing in the East part of the city were more often satisfied with their neighbourhood than male respondents, respondents with a low socio-economic status and respondents residing in the West part of the city. The stratification by place of residence showed the most pronounced difference. Among the respondents residing in the West part of the city, about one third was satisfied with their neighbourhood, while among the respondents residing in the East part of the city, about two thirds were satisfied with their neighbourhood (p < 0.05), even though the walkability measures between the East and the West part of the city did not differ from each other (data not shown).

**Table 1:** Descriptive characteristics of the sample

		N	General neighbourhood satisfaction	Socio-environmental quality	Social cohesion	Local infrastructure
			% Satisfied			
Sex	Male	401	46.1 %	47.4 %	41.2 %*	47.0 %
	Female	442	53.9 %	52.6 %	52.4 %*	53.0 %
Age	15–19	85	11.2 %	11.2 %	11.4 %	9.9 %
	20–24	80	9.1 %	9.3 %	7.9 %	8.0 %
	25–29	107	11.0 %	11.9 %	9.8 %	13.3 %
	30–34	83	8.6 %	9.3 %	9.8 %	11.2 %
	35–39	108	13.6 %	12.9 %	12.9 %	14.4 %
	40–44	101	13.1 %	12.4 %	11.9 %	12.8 %
	45–49	97	11.2 %	11.0 %	11.9 %	8.7 %
	50–54	80	9.7 %	10.5 %	10.5 %	10.6 %
	55–60	102	12.5 %	11.7 %	14.0 %	11.0 %
Socio-economic status	Low	422	47.6 %	45.5 %*	49.5 %	52.1 %
	High	421	52.4 %	54.5 %*	50.5 %	47.9 %
Place of residence	West	334	30.6 %*	27.6 %*	34.5 %*	36.2 %*
	East	509	69.4 %*	72.4 %*	65.5 %*	63.8 %*
			Median (IQR)			
Gross population density			4424.7 (4802.9)			
Household unit density			1998.3 (2473.8)			
Entropy index			0.75 (0.17)			
Proportion of mixed land use			50.1 (44.6)			
Three-way intersection density			86.1 (32.4)			
Four-way intersection density			14.3 (15.5)			
IPEN walkability index			0.18 (3.17)			
Graz walkability index			−0.25 (4.43)			

*p < 0.05

Tables 2 and 3 show the results on the association between each walkability measure and neighbourhood satisfaction. Gross population density, household unit density, proportion of mixed land use and four-way intersection density were negatively associated with all neighbourhood satisfaction factors, except with satisfaction with local infrastructure, where the association was positive. The entropy index and three-way intersection density were less consistently associated with neighbourhood satisfaction.

**Table 2:** Results from the logistic regression on the associations between walkability measures and general neighbourhood satisfaction

		General neighbourhood satisfaction^a^	
		Men	Women
Gross population density	OR (95 % CI)	0.7 (0.6 0.9)	0.6 (0.4 0.7)
	p-value	0.001*	0.000*
	Nagelkerke r^2^	0.110	0.212
Household unit density	OR (95 % CI)	0.7 (0.6 0.9)	0.6 (0.4 0.7)
	p-value	0.002*	0.000*
	Nagelkerke r^2^	0.110	0.212
Entropy index	OR (95 % CI)	1.0 (0.8 1.2)	1.1 (0.9 1.3)
	p-value	0.642	0.577
	Nagelkerke r^2^	0.079	0.131
% Mixed land use	OR (95 % CI)	0.7 (0.6 0.9)	0.6 (0.5 0.7)
	p-value	0.005*	0.000*
	Nagelkerke r^2^	0.103	0.193
Three-way intersection density	OR (95 % CI)	1.0 (0.7 1.1)	0.8 (0.7 1.0)
	p-value	0.436	0.084
	Nagelkerke r^2^	0.080	0.138
Four-way intersection density	OR (95 % CI)	0.8 (0.7 1.0)	0.7 (0.6 0.9)
	p-value	0.042*	0.001*
	Nagelkerke r^2^	0.091	0.162
IPEN walkability index	OR (95 % CI)	0.9 (0.9 1.0)	0.9 (0.8 1.0)
	p-value	0.067	0.004*
	Nagelkerke r^2^	0.089	0.153
Graz walkability index	OR (95 % CI)	0.9 (0.8 1.0)	0.8 (0.8 0.9)
	p-value	0.004*	0.000*
	Nagelkerke r^2^	0.104	0.196

*p < 0.05

^a^Adjusted for socio-economic status, age and place of residence

**Table 3:** Results from the logistic regression on the associations between walkability measures and neighbourhood satisfaction indicators

		Socio-environmental quality^a^		Social cohesion^a^		Local infrastructure^a^	
		Men	Women	Men	Women	Men	Women
Gross population density	OR (95 % CI)	0.5 (0.4 0.6)	0.4 (0.3 0.5)	0.7 (0.6 0.9)	0.6 (0.5 0.8)	1.5 (1.2 1.9)	1.3 (1.1 1.6)
	p-value	0.000*	0.000*	0.002*	0.000*	0.000*	0.012*
	Nagelkerke r^2^	0.231	0.267	0.074	0.134	0.092	0.084
Household unit density	OR (95 % CI)	0.5 (0.4 0.6)	0.4 (0.3 0.5)	0.7 (0.6 0.9)	0.6 (0.5 0.8)	1.5 (1.2 1.9)	1.3 (1.5 1.6)
	p-value	0.000*	0.000*	0.002*	0.000*	0.000*	0.015*
	Nagelkerke r^2^	0.230	0.269	0.073	0.135	0.092	0.082
Entropy index	OR (95 % CI)	0.8 (0.7 1.0)	0.7 (0.6 0.9)	1.0 (0.8 1.2)	0.9 (0.7 1.1)	1.4 (1.1 1.7)	1.5 (1.2 1.9)
	p-value	0.083	0.006*	0.899	0.154	0.003*	0.000*
	Nagelkerke r^2^	0.128	0.130	0.041	0.083	0.072	0.109
% Mixed land use	OR (95 % CI)	0.5 (0.4 0.6)	0.4 (0.3 0.5)	0.7 (0.6 0.9)	0.7 (0.6 0.8)	1.6 (1.3 2.0)	1.3 (1.1 1.6)
	p-value	0.000*	0.000*	0.001*	0.000*	0.000*	0.008*
	Nagelkerke r^2^	0.227	0.283	0.076	0.120	0.100	0.086
Three-way intersection density	OR (95 % CI)	0.7 (0.6 1.0)	0.8 (0.6 0.9)	0.8 (0.6 1.0)	0.8 (0.7 1.0)	1.4 (1.1 1.7)	1.5 (1.2 1.8)
	p-value	0.008*	0.008*	0.022*	0.055	0.003*	0.000*
	Nagelkerke r^2^	0.140	0.128	0.058	0.088	0.071	0.108
Four-way intersection density	OR (95 % CI)	0.6 (0.5 0.8)	0.6 (0.5 0.7)	0.7 (0.6 0.9)	0.7 (0.6 0.9)	1.6 (1.3 2.0)	1.4 (1.2 1.7)
	p-value	0.000*	0.000*	0.004*	0.004*	0.000*	0.001*
	Nagelkerke r^2^	0.166	0.171	0.068	0.101	0.097	0.098
IPEN walkability index	OR (95 % CI)	0.8 (0.8 0.9)	0.8 (0.8 0.9)	0.9 (0.8 1.0)	0.9 (0.8 1.0)	1.2 (1.1 1.3)	1.2 (1.1 1.3)
	p-value	0.000*	0.000*	0.008*	0.001*	0.000*	0.000*
	Nagelkerke r^2^	0.184	0.189	0.064	0.109	0.109	0.131
Graz walkability index	OR (95 % CI)	0.8 (0.7 0.9)	0.7 (0.7 0.8)	0.9 (0.8 1.0)	0.9 (0.8 0.9)	1.2 (1.1 1.3)	1.1 (1.0 1.2)
	p-value	0.000*	0.000*	0.001*	0.000*	0.000*	0.003*
	Nagelkerke r^2^	0.217	0.256	0.077	0.124	0.104	0.092

*p < 0.05

^a^Adjusted for socio-economic status, age and place of residence

The odds of being generally satisfied with the neighbourhood decreased by 10–30 % with each unit increase in walkability. Overall, the factors included in the model explained 8–21 % of the variation in general neighbourhood satisfaction.

Comparing the strength of association across all neighbourhood satisfaction measures, the association between walkability and socio-environmental quality was the strongest. The odds of being satisfied with socio-environmental quality decreased by 20–60 % with each unit increase in walkability. Connectivity showed the weakest association with satisfaction with socio-environmental quality. Nevertheless, the odds of being satisfied with the socio-environmental quality decreased by 20–40 % with each unit increase in intersectioin density. Overall, the factors included in the model explained about 13–28 % of the variation in satisfaction with socio-environmental quality.

The association between walkability and satisfaction with social cohesion was less strong. The odds of being satisfied with social cohesion decreased by 10–40 % for each unit increase in walkability. Overall, the factors included in the model explained about 4–13 % of the variation in satisfaction with social cohesion.

Unlike the other neighbourhood satisfaction measures, walkability was positively associated with satisfaction with the local infrastructure. The odds of being satisfied with the local infrastructure increased by 20–60 % with each unit increase in walkability. All statistically significantly associated walkability measures showed similarly strong associations with satisfaction with the local infrastructure. Overall, the factors included in the model explained about 7–13 % of the satisfaction with the local infrastructure.

In many cases, walkability showed a slightly stronger association with neighbourhood satisfaction among women than among men.

## Discussion

Based on the theory of Frumkin et al. (2004), it was hypothesised that walkability would be positively associated with neighbourhood satisfaction. As expected, walkability was positively associated with satisfaction with local infrastructure. High-walkability neighbourhoods have per definition a higher land use mix. Therefore, local infrastructure measured objectively should be better than in low-walkability, more residential areas. Consequently, the odds of being satisfied with the infrastructure increased with improved walkability.

Contrary to expectations, the walkability factors investigated in the present study were negatively associated with general neighbourhood satisfaction, satisfaction with socio-environmental quality and satisfaction with social cohesion. Neighbourhood satisfaction decreased with an increase in walkability.

Our study confirms evidence on a negative association between walkability and neighbourhood satisfaction and social capital (Hanibuchi et al. 2012; Van Dyck et al. 2011; Van Dyck et al. 2013; Wood et al. 2010; Wood et al. 2008). This negative association might be explained by other issues related to high-walkability areas that confound the association of walkability with neighbourhood satisfaction and social capital. In Belgium, for instance, these negative associations were mediated by perceived environmental characteristics, such as aesthetics and safety (Van Dyck et al. 2011). Also, Howley et al. (2009) argued that factors such as safety, noise, traffic, pollution, etc., might have an impact on the negative association between density and neighbourhood satisfaction (Howley et al. 2009). French et al. (2014) supported this argumentation by showing that density and perceived safety were negatively associated with sense of community, while perceived aesthetic quality was positively associated with sense of community (French et al. 2014).

It could also be that there is a threshold of density, land use mix, connectivity and walkability beyond which neighbourhood satisfaction declines (Wood et al. 2010; Wood et al. 2008). Wood et al. (2008) found a negative association between number of destinations and social capital and argued that an optimum number and mix of destinations might be necessary to improve social capital. The same might apply to neighbourhood satisfaction. Moudon et al. (2006) tried to estimate threshold values for density or destinations in relation to walking (Moudon et al. 2006). So far, no study has attempted to use such threshold values to explore the association between walkability and neighbourhood satisfaction.

Furthermore, the socio-economic composition of the population might also have an impact. Areas with a low population density and therefore low walkability are usually more affluent (McCulloch 2003). The socio-economic status on the neighbourhood level could confound the association between walkability and neighbourhood satisfaction. Sallis et al. (2009) investigated the difference in associations between walkability and neighbourhood satisfaction by neighbourhood income. They found a positive association between walkability and neighbourhood satisfaction, but only among respondents living in high-income neighbourhoods. A similar phenomenon was observed when we stratified our results by socio-economic status and place of residence, but on an individual level. The negative association between walkability and neighbourhood satisfaction with social cohesion and the positive association between walkability and neighbourhood satisfaction with infrastructure tended to be stronger and more frequent among high socio-economic groups and among residents living in the eastern part of the city (Grasser 2014). In general, individual socio-economic status and place of residence were important confounding factors throughout our analysis (Grasser 2014; Grasser et al. 2014). Therefore, the neighbourhood socio-economic status also could confound the results of our study. More research needs to be done to investigate this issue further.

From all the walkability indicators used in our study, the entropy index and three-way intersection density were least associated with neighbourhood satisfaction. Since the zoning data in our study did not distinguish well between different land use types, the entropy index did not provide a valid measure in our case (Grasser et al. submitted). Additionally, there are other issues related to the entropy index (Grasser et al. submitted), and the question remains whether simpler measures of land use mix (e.g. the proportion of mixed land use) are more valid measures (Lee and Moudon 2006; Cerin et al. 2007; Grasser et al. submitted). In our study, four-way intersection density was consistently associated with neighbourhood satisfaction, while three-way intersection density only showed associations in individual cases. In a relatively high-walkability city such as Graz, four-way intersection density might be the more valid measure because it differentiates better between high- and low-walkability areas (Grasser et al. submitted).

Associations between walkability and health are sensitive to the geographical scale of the neighbourhood (Diez Roux 2007). Using a circular buffer of 1000 m and a street network buffer of 1500 m showed the same pattern of association as reported here (Grasser 2014). Some authors argue that in a European context a larger neighbourhood might better represent the characteristics of the neighbourhood (Grasser 2014; Grasser et al. submitted; Oliver et al. 2007).

To our knowledge, no study so far has stratified the analysis by sex when looking at the association between neighbourhood characteristics and neighbourhood satisfaction or social capital. In our study, the associations between walkability and neighbourhood satisfaction were slightly stronger among women than among men. Women usually have different roles and tasks than men and have to meet the requirements of being in a professional job and of being a mother. Since they are more involved in child care, schooling, etc., than men, they have to develop social networks in the community around these tasks (McCulloch 2003). Therefore, women, and especially those with children, show a higher sense of community and a higher level of social capital (Du Toit et al. 2007; McCulloch 2003). Assuming that there is a relationship between neighbourhood characteristics and neighbourhood satisfaction, it would make sense that this relationship would be stronger for women than for men.

There are some limitations that have to be taken into account. Due to the cross-sectional design of the study, causality cannot be established. The street centre line data were not in the best possible condition. However, using a combination of different operations during the modelling process enabled a valid analysis (Grasser 2014). The results of the study are based on sound theoretical and empirical evidence, which was ensured by a systematic literature review (Grasser et al. 2013), the use of a reliable questionnaire (Titze et al. 2007), the representativeness of the sample (Titze et al. 2008) and the objective walkability measures.

In conclusion, our study has shown that walkability seems to be negatively associated with most neighbourhood satisfaction measures (except satisfaction with infrastructure) in the city of Graz. The results contribute to the current limited understanding of the associations between walkability and neighbourhood satisfaction and sociability, especially in a European context. However, additional, more comparable, longitudinal research is desirable to determine what impact walkability has on neighbourhood satisfaction and to identify the important mediating factors.
